# Long non‐coding RNA LINC01535 promotes cervical cancer progression via targeting the miR‐214/EZH2 feedback loop

**DOI:** 10.1111/jcmm.14476

**Published:** 2019-07-05

**Authors:** Hongjuan Song, Yuan Liu, Xin Jin, Yang Liu, Yanling Yang, Lei Li, Xuan Wang, Guilin Li

**Affiliations:** ^1^ Department of Gynecology Xuzhou Maternal & Child Health Care Hospital Xuzhou China

**Keywords:** cervical cancer, feedback loop, long non‐coding RNA, microRNA, progression

## Abstract

Long non‐coding RNAs (lncRNAs) have shown critical roles in multiple cancers via competitively binding common microRNAs. miR‐214 has been proved to play tumour suppressive roles in various cancers, including cervical cancer. In this study, we identified that lncRNA LINC01535 physically binds miR‐214, relieves the repressive roles of miR‐214 on its target EZH2, and therefore up‐regulates EZH2 protein expression. Intriguingly, we also found that EZH2 directly represses the expression of *miR‐214*. Thus, miR‐214 and EZH2 form double negative regulatory loop. Through up‐regulating EZH2, LINC01535 further represses *miR‐214* expression. Functional experiments showed that enhanced expression of LINC01535 promotes cervical cancer cell growth, migration and invasion in vitro and cervical cancer xenograft growth in vivo. Reciprocally, LINC01535 knockdown suppresses cervical cancer cell growth, migration and invasion. Activation of the miR‐214/EZH2 regulatory loop by overexpression of miR‐214 or silencing of EZH2 reverses the roles of LINC01535 in promoting cervical canc`er cell growth, migration and invasion in vitro and cervical cancer xenograft growth in vivo. Clinically, LINC01535 is significantly up‐regulated in cervical cancer tissues and correlated with advanced clinical stage and poor prognosis. Moreover, the expression of LINC01535 is reversely associated with the expression of miR‐214 and positively associated with the expression of EZH2 in cervical cancer tissues. In conclusion, this study reveals that LINC01535 promotes cervical cancer progression via repressing the miR‐214/EZH2 regulatory loop.

## 1 INTRODUCTION

According to global cancer statistics 2018, the incidence and mortality of cervical cancer both rank fourth among cancers in female patients, with 5 69 847 new cervical cancer cases and 3 11 365 deaths in 2018, globally.^1^ Although great advances have been achieved in therapeutic strategies against cervical cancer, including surgical resection, radiotherapy and chemotherapy, the long‐term prognosis of cervical cancer patients is still unsatisfactory because of frequent post‐surgical recurrence and/or resistance to radiotherapy and chemotherapy.^2^ Recently, molecular targeted therapies have greatly improved the outcome of many cancers, such as melanoma, breast cancer, lung cancer and prostate cancer.^3^, ^4^ However, most cervical cancers are not sensitive to currently available molecular targeted therapies.^5^ Therefore, further enhancing our understanding of molecular mechanisms underlying the initiation and progression of cervical cancer is beneficial for developing more effective treatments for cervical cancer.^6^

Transcriptome sequencings have found many deregulated non‐coding RNAs in cancers.^7^, ^8^ Concurrently, increasingly reports have shown the important roles of non‐coding RNAs in multiple cancers.^9^, ^10^, ^11^ Among these non‐coding RNAs, microRNAs and long non‐coding RNAs are two main classes. microRNAs (miRNAs) are a class of short RNAs with a length of 21‐25 nucleotides.^12^, ^13^, ^14^, ^15^, ^16^ Long non‐coding RNAs (lncRNAs) are a class of long RNAs with lengths of more than 200 nucleotides and limited protein coding potential.^17^, ^18^, ^19^, ^20^ miRNAs are well known to induce translation repression and/or degradation of their target messenger RNAs (mRNAs) via base pairing at partially or fully complementary sites.^21^, ^22^, ^23^, ^24^ The function manners of lncRNAs are complex and various.^25^, ^26^, ^27^, ^28^ One of the action mechanisms of lncRNAs is to physically bind and sequester miRNAs and relieve the repressive roles of miRNAs on their genuine target mRNAs.^29^, ^30^, ^31^ These lncRNAs are also known as competitive endogenous RNAs (ceRNAs).^32^, ^33^

In our previous study, we have identified that miR‐214 is down‐regulated in human cervical cancer and significantly inhibits cervical cancer growth.^34^ Other reports also showed the tumour suppressive roles of miR‐214 in cervical cancer via modulating cell survival, cell migration, cell invasion, drug sensitive and so on.^35^, ^36^, ^37^, ^38^, ^39^ Except for cervical cancer, miR‐214 was also reported by different authors to act as tumour suppressor in multiple cancers, including colorectal cancer, oesophageal squamous cell carcinoma, breast cancer, papillary thyroid carcinoma and so on.^37^, ^40^, ^41^, ^42^ Because several lncRNAs are revealed to bind and sequester miRNAs and block the roles of miRNAs, we hypothesized that there are lncRNAs, which bind and sequester miR‐214 and further exert oncogenic roles in cervical cancer.

In this study, we performed an unbiased screen to search the lncRNAs which could bind miR‐214 and finally identify lncRNA LINC01535 as a ceRNA for miR‐214. We investigated the expression and roles of LINC01535, and resolved in detail, the mechanisms of action of LINC01535 in cervical cancer.

## 2 MATERIALS AND METHODS

### 2.1 Cell culture

Human cervical cancer cell lines HeLa, SiHa and CaSki were obtained from the American Type Culture Collection (Manassas, VA, USA). HeLa and SiHa cells were maintained in Eagle's Minimum Essential Medium (MEM) (Invitrogen, Carlsbad, CA, USA). CaSki cells were maintained in RPMI‐1640 Medium (Invitrogen). All the cells were cultured in the medium supplemented with 10% foetal bovine serum (Invitrogen) at 37°C with 5% CO_2_.

### 2.2 Plasmids construction

The 3’ 630 nucleotides of LINC01535 containing the predicted miR‐214 binding sites and the 3’UTR of EZH2 containing the reported miR‐214 binding sites were PCR‐amplified with the Platinum^®^ *Pfx* DNA Polymerase (Invitrogen) and the primers 5'‐CGAGCTCCTGTGGGGATGGAAGTGTGA‐3' (sense) and 5'‐GCTCTAGATGGGAGGGAGATAAGGAAAATG‐3' (antisense) for LINC01535, or 5'‐CGAGCTCGAAATCCCTTGACATCTGC‐3' (sense) and 5'‐GCTCTAGAGTTGAAAAATGTACCATACTGC‐3' (antisense) for EZH2, respectively. The PCR products were then cloned into the Sac I and Xba I sites of pmirGLO plasmid (Promega, Madison, WI, USA), named as pmirGLO‐LINC01535 or pmirGLO‐EZH2, respectively. The complementary DNA (cDNA) encoding LINC01535 was PCR‐amplified with the Platinum^®^ *Pfx* DNA Polymerase (Invitrogen) and the primers 5'‐GGAATTCAGCCCGGCGGACGCTGGGT‐3' (sense) and 5'‐GCTCTAGAGGTTAATTTGATTCTCATTCCAC‐3' (antisense). The PCR products were then cloned into the EcoR I and Xba I sites of pcDNA™3.1(+) plasmid (Invitrogen), named as pcDNA‐LINC01535. EZH2 overexpression plasmid was purchased from FulenGen (Guangzhou, China) (Catalog# EX‐Z0388‐M02). The cDNA oligonucleotides repressing LINC01535 expression were synthesized by GenePharma (Shanghai, China) and inserted into the GenePharma SuperSilencing™ shRNA expression vector pGPU6/Hygro, named as sh‐LINC01535. The shRNA target sites were 5'‐GGAAGTGTGATTGCTTCATTC‐3'. EZH2 specific shRNA was purchased from FulenGen (Guangzhou, China) (Catalog# HSH095626‐nU6). miR‐214 mimics and inhibitors and their respective negative controls (NC) were purchased from Applied Biosystems (Foster City, CA, USA). The transfections of plasmids and miRNAs were carried out with the Lipofectamine 3000 (Invitrogen) following the protocol.

### 2.3 Dual luciferase reporter assay

pmirGLO or pmirGLO‐LINC01535 was co‐transfected with miR‐214 mimics or miR‐NC into HeLa cells. pmirGLO or pmirGLO‐LINC01535 was co‐transfected with miR‐214 inhibitors or inh‐NC into HeLa cells. pmirGLO or pmirGLO‐EZH2, pcDNA or pcDNA‐LINC01535, and miR‐214 mimics or miR‐NC were co‐transfected into HeLa cells. pmirGLO or pmirGLO‐EZH2 was co‐transfected with sh‐LINC01535 or sh‐NC into HeLa cells. After culturing for 48 hours, the Firefly luciferase activity and Renilla luciferase activity were detected with the Dual‐Luciferase^®^ Reporter Assay System (Promega) following the manufacturer's instructions.

### 2.4 Isolation of cytoplasmic and nuclear RNA

Cytoplasmic and nuclear RNA from HeLa cells was isolated using the Cytoplasmic & Nuclear RNA Purification Kit (Norgen, Belmont, CA, USA) following the manufacturer's instruction. The gene expression for specific genes was measured using qRT‐PCR.

### 2.5 RNA isolation and quantitative real‐time polymerase chain reaction (qRT‐PCR)

RNA was extracted using the TRIzol Reagent (Invitrogen) following the manufacturer's instruction. Next, reverse transcription was carried out using the extracted RNA and PrimeScript™ II 1st Strand cDNA Synthesis Kit (Takara, Dalian, China) to generate first‐stand cDNA. For the quantification of LINC01535 expression, quantitative real‐time polymerase chain reaction (qRT‐PCR) was carried out using SYBR^®^ Premix Ex Taq™ II kit (Takara) on 7500 Real‐Time PCR System (Applied Biosystems) following the standard SYBR Green protocol. Primers sequences were as follows: for LINC01535, 5'‐GGGATGGAAGTGTGATTGC‐3' (sense) and 5'‐TGATGCTAGGGGTGCTAAG‐3' (antisense); for GAPDH, 5'‐GGTCTCCTCTGACTTCAACA‐3' (sense) and 5'‐GTGAGGGTCTCTCTCTTCCT‐3' (antisense). GAPDH was employed as an endogenous control for the quantification of LINC01535 expression. For the quantification of miRNAs, qRT‐PCR was carried out using TaqMan microRNA assays (Applied Biosystems) on 7500 Real‐Time PCR System (Applied Biosystems) following the manufacturer's protocol. The expression of RNAs was calculated following the comparative Ct method.

### 2.6 RNA pull‐down

LINC01535 was in vitro transcribed and biotin‐labelled from pSPT19‐LINC01535 with the Biotin RNA Labeling Mix (Roche) and Sp6 RNA polymerase (Roche) following the manufacturer's instructions. After being treated with DNase I (Takara), the in vitro transcribed RNAs were purified with the RNeasy Mini Kit (Qiagen, Valencia, CA, USA) following the manufacturer's instructions. Next, 3 µg of purified biotin‐labelled RNAs were incubated with 1 mg of HeLa cell lysates at 25°C for 1 hour. The complexes were isolated with streptavidin agarose beads (Invitrogen). The miRNAs enriched in the pull‐down material were measured by qRT‐PCR as above described.

### 2.7 Western blot

Total cell lysates were extracted from transfected cells 48 hours after transfection or stable cell lines with RIPA lysis buffer (Beyotime, Shanghai, China) added with protease inhibitors (Beyotime) in accordance with the instructions. Equal quantities of proteins were separated by 10% sodium dodecyl sulphate‐polyacrylamide gel electrophoresis, followed by transfer to polyvinylidene difluoride membrane (Millipore, Billerica, MA, USA). The membranes were incubated with 5% non‐fat milk for 90 minutes at room temperature. Next, the membranes were incubated with primary antibodies against EZH2 (Cell Signaling Technology, Danvers, MA, USA) or GAPDH (Cell Signaling Technology) overnight at 4°C. The membranes were further incubated with IRdye 700‐conjugated goat anti‐mouse IgG or IRdye 800‐conjugated goat anti‐rabbit IgG second antibodies (Invitrogen) and detected on an Odyssey infrared scanner (Li‐Cor, Lincoln, NE, USA). GAPDH was employed as an endogenous control for the quantification of EZH2 protein expression.

### 2.8 Chromatin immunoprecipitation (ChIP)

Chromatin immunoprecipitation (ChIP) was performed in HeLa cells using the EZ‐Magna ChIP™ A/G Chromatin Immunoprecipitation Kit (Millipore) and EZH2 specific antibody (Millipore) following the manufacturer's instructions. The enriched DNA was measured by qRT‐PCR using SYBR^®^ Premix Ex Taq™ II kit (Takara) on 7500 Real‐Time PCR System (Applied Biosystems) following the standard SYBR Green protocol. The sequences of primers corresponding to the promoter of *miR‐214* were as follows: 5'‐ATGTCTGAGAGCAGGCGATT‐3' (sense) and 5'‐TAGGACCAGGAAAAGGGGG‐3' (antisense).

### 2.9 Stable cell lines construction

To stably overexpress LINC01535 in HeLa and SiHa cells, pcDNA‐LINC01535 or pcDNA was transfected into HeLa and SiHa cells. Seventy‐two hours after transfection, the cells were selected with neomycin for 4 weeks. To stably silence LINC01535 in HeLa and CaSki cells, sh‐LINC01535 or sh‐NC was transfected into HeLa and CaSki cells. Seventy‐two hours after transfection, the cells were selected with hygromycin for 4 weeks. To obtain LINC01535 and miR‐214 concurrently stably overexpressed cells, LINC01535 stably overexpressed HeLa cells were transfected with 2 × 10^6^ transducing units of miR‐214 overexpression lentiviruses (FulenGen, Guangzhou, China). Seventy‐two hours after transfection, the cells were selected with puromycin for 4 weeks. To obtain LINC01535 stably overexpressed and concurrent EZH2 stably silenced cells, sh‐EZH2 was transfected into LINC01535 stably overexpressed HeLa cells. Seventy‐two hours after transfection, the cells were selected with puromycin for 4 weeks.

### 2.10 Cell viability assay and Ethynyl deoxyuridine (EdU) staining assay

Cell growth ability was evaluated by Glo cell viability assay and Ethynyl deoxyuridine (EdU) staining assay. For Glo cell viability assay, 3000 indicated cervical cancer cells were seeded into 96‐well plates per well. After culture for indicated time, cell viabilities were measured using the CellTiter‐Glo^®^ Luminescent Cell Viability Assay (Promega) in accordance with the manufacturer's protocol. EdU staining was performed using the EdU kit (RiboBio, Guangzhou, China) in accordance with the manufacturer's protocol. The percentage of EdU positive cells was evaluated using Zeiss Photomicroscope (Carl Zeiss, Oberkochen, Germany) and counted dependent on at least five random fields.

### 2.11 Transwell migration and invasion assays

Cell migration and invasion ability was evaluated by transwell migration and invasion assays. Forty thousand indicated cervical cancer cells re‐suspended in serum‐free medium were plated into the 24‐well transwell chambers (Millipore) per well. For invasion assay, the transwell chambers were pre‐coated with Matrigel (BD Biosciences, San Jose, CA, USA). Complete medium containing 10% foetal bovine serum was added into the lower chamber. After incubation for 48 hours, the non‐migratory and non‐invasive cells at upper chambers were removed using cotton‐tipped swabs. The migratory and invasive cells at the lower surfaces of chambers were fixed using methanol and stained using 0.1% crystal violet solution. The counting of the migratory and invasive cells was performed using Zeiss Photomicroscope dependent on at least five random fields.

### 2.12 Xenograft assay in mice

Five‐six‐week‐old female BALB/c‐nu/nu nude mice were purchased from SLRC Laboratory Animal Center (Shanghai, China) and bred in the pathogen‐free condition. 5 × 10^6^ indicated cervical cancer cells were subcutaneously injected into the flanks of the nude mice. The volumes of subcutaneous tumours were measured every four days with a calliper, and calculated according to the equation V = a × b^2^/2 (a, long axes; b, short axes). On the 24th day after inoculation, subcutaneous tumours were resected and weighed. The xenograft assays were approved by the Ethics Committee of the Xuzhou Maternity & Child Health Care Hospital (Xuzhou, China).

### 2.13 Clinical specimens

A total of 80 pairs of cervical cancer tissues and matched adjacent normal cervical tissues were acquired from cervical cancer patients with written informed consent at the Xuzhou Maternity & Child Health Care Hospital (Xuzhou, China). All specimens were examined by pathologists. This study was carried out according to the principles of Declaration of Helsinki and approved by the Ethics Committee of the Xuzhou Maternity & Child Health Care Hospital (Xuzhou, China).

### 2.14 Immunohistochemistry (IHC) staining

Quantification of EZH2 expression in cervical cancer tissues was performed using immunohistochemistry (IHC) staining as previously described with an EZH2 specific antibody (Cell Signaling Technology). Quantification of Ki67 and cleaved caspase‐3 expression in subcutaneous tumours was performed using IHC staining with specific antibodies against Ki67 (Abcam, Hong Kong, China) or cleaved caspase‐3 (Cell Signaling Technology).

### 2.15 Statistical analysis

SPSS 18.0 software package (Chicago, IL, USA) was used to carry out statistical analyses. For comparisons, Student's *t* test, one‐way ANOVA followed by Dunnett's multiple comparison test, Kruskal‐Wallis test followed by Dunnett's multiple comparison test, Wilcoxon signed‐rank test, Pearson chi‐square test, Log‐rank test, Pearson correlation analysis and Mann‐Whitney test were performed as indicated. *P* value < 0.05 was considered as statistically significant.

## 3 RESULTS

### 3.1 LINC01535 physically binds miR‐214

**Figure 1:**
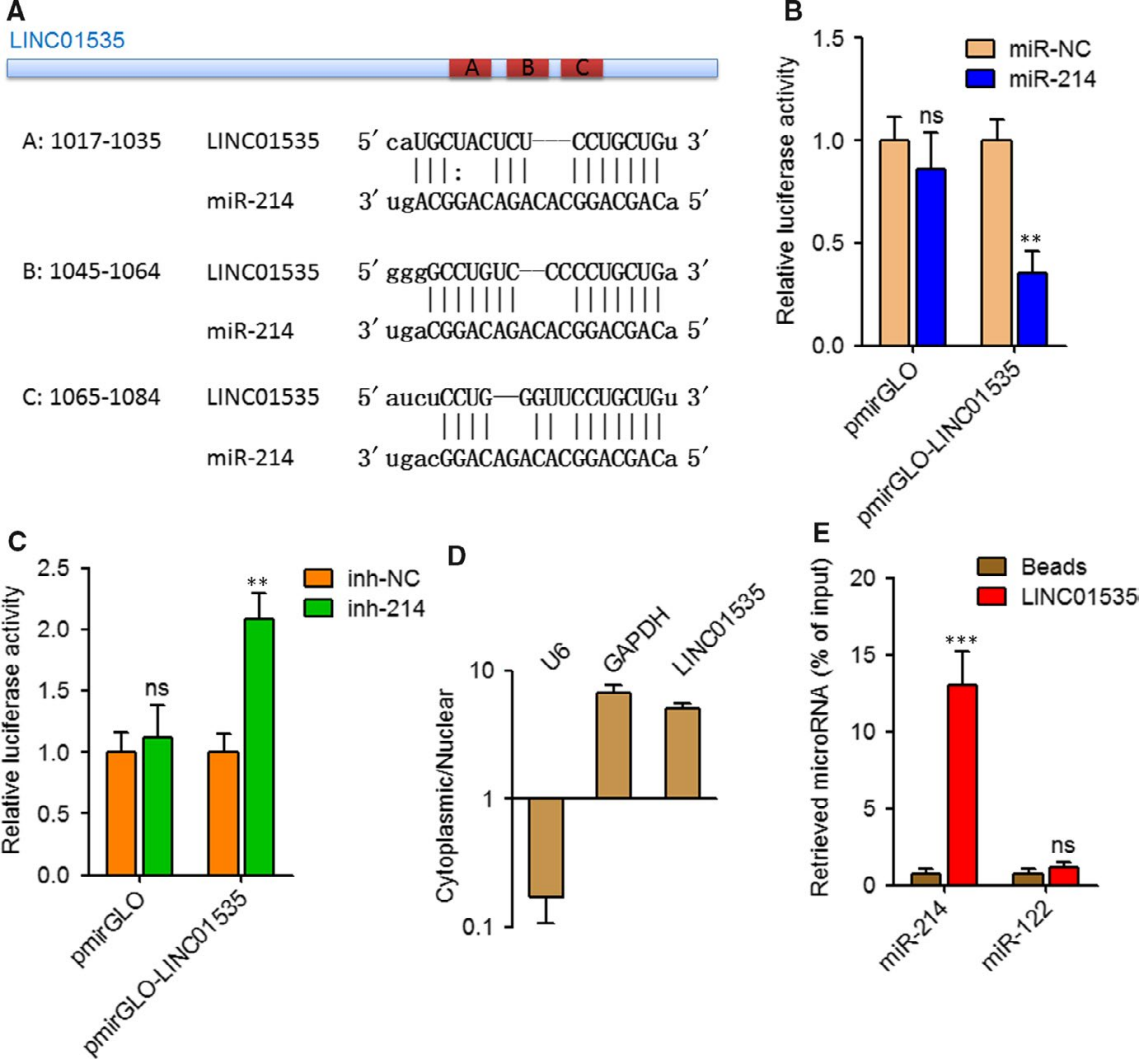
LINC01535 interacts with miR‐214. A, Schematic diagram of the predicted miR‐214 binding sites on LINC01535. B, Dual luciferase reporter assay in HeLa cells co‐transfected with luciferase reporter containing LINC01535 or nothing and miR‐214 mimics or miRNA negative control (miR‐NC). Results are displayed as the relative ratio of Firefly luciferase activity to Renilla luciferase activity. C, Dual luciferase reporter assay in HeLa cells co‐transfected with luciferase reporter containing LINC01535 or nothing and miR‐214 inhibitors or inhibitor negative control (inh‐NC). Results are displayed as the relative ratio of Firefly luciferase activity to Renilla luciferase activity. D, The levels of LINC01535 in cytoplasmic or nuclear RNAs purified from Huh7 cells. U6 and GAPDH serve as nuclear and cytoplasmic control, respectively. E, HeLa cell lysates were incubated with biotin‐labeled LINC01535; after pull‐down, miRNAs was extracted and detected by qRT‐PCR. Results are shown as mean ± SD of three independent experiments. **P < 0.01, ***P < 0.001, ns, not significant, by Student's *t* test

First, we predicted 256 miR‐214 binding sites on 209 lncRNAs using the starBase^43^ (http://starbase.sysu.edu.cn/agoClipRNA.php?source=lncRNA) (Table S1). Notably, one of these predicted lncRNAs, LINC01535 has three consecutive miR‐214 binding sites in a span of 68 nucleotides (Figure 1A), which indicates strong possibility of miRNA‐lncRNA binding. Next, the 3’ 630 nucleotides of LINC01535 containing the predicted miR‐214 binding sites were cloned into luciferase reporter pmirGLO. Dual luciferase reporter assays displayed that enhanced expression of miR‐214 significantly repressed the luciferase activity of constructed reporter but not empty reporter (Figure 1B). Reciprocally, inhibition of miR‐214 significantly increased the luciferase activity of constructed reporter but not empty reporter (Figure 1C). Subcellular distribution of LINC01535 in cervical cancer cells was detected. As displayed in Figure 1D, LINC01535 was mainly located in cytoplasm, which supports the potential binding between LINC01535 and miRNAs. To validate the direct binding between LINC01535 and miR‐214, affinity pull‐down of endogenous miR‐214 by in vitro transcribed biotinylated LINC01535 was carried out. As displayed in Figure 1E, miR‐214 was specifically enriched by LINC01535. Therefore, these data demonstrated that LINC01535 physically binds miR‐214.

### 3.2 LINC01535 represses the miR‐214/EZH2 regulatory loop

**Figure 2:**
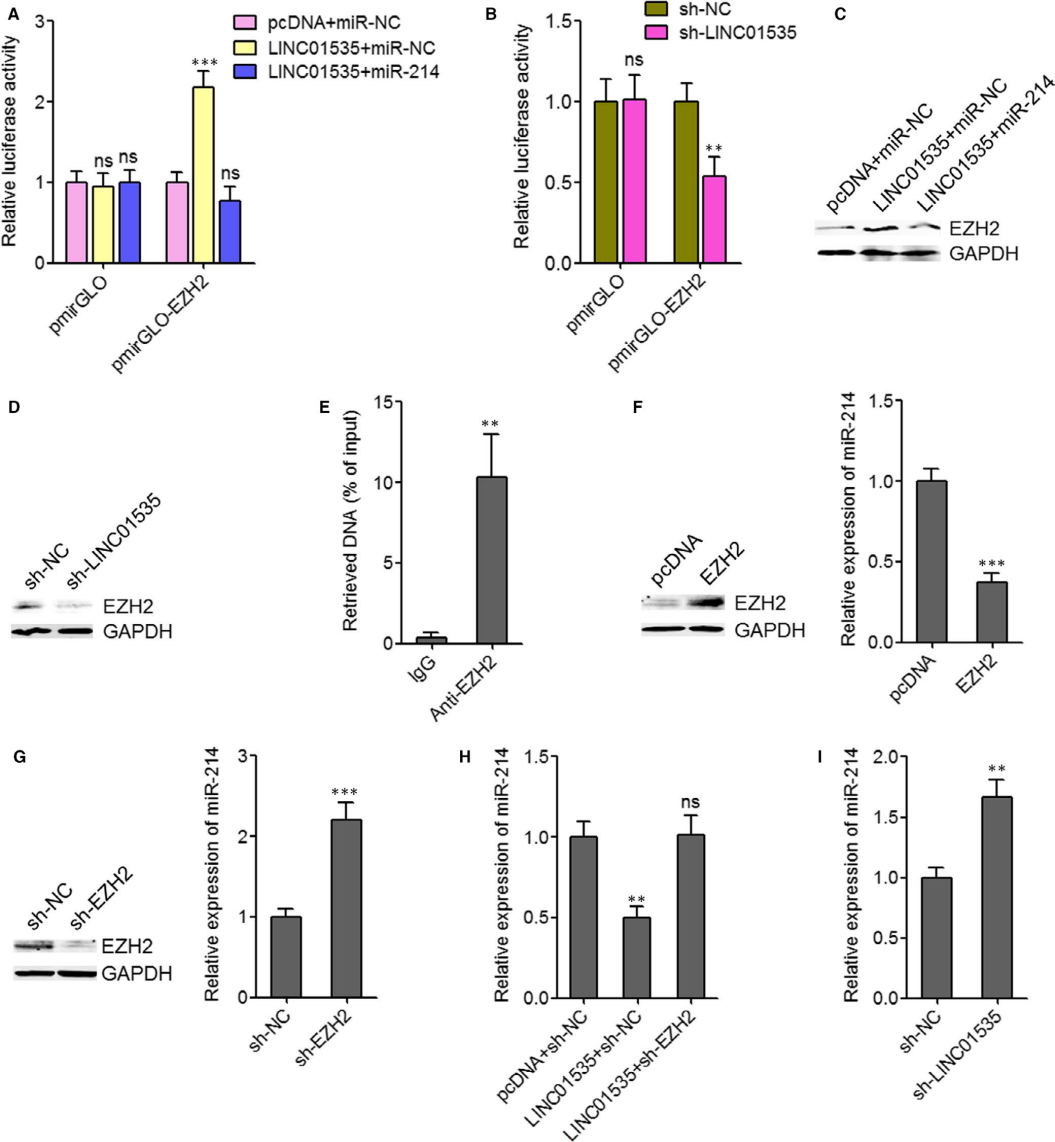
LINC01535 represses the miR‐214/EZH2 regulatory axis. A, Dual luciferase reporter assay in HeLa cells co‐transfected with luciferase reporter containing EZH2 3’UTR or nothing, LINC01535 overexpression plasmid (pcDNA‐LINC01535) or empty plasmid (pcDNA), and miR‐214 mimics or miR‐NC. Results are displayed as the relative ratio of Firefly luciferase activity to Renilla luciferase activity. B, Dual luciferase reporter assay in HeLa cells co‐transfected with luciferase reporter containing EZH2 3’UTR or nothing and LINC01535 specific shRNA (sh‐LINC01535) or negative control shRNA (sh‐NC). Results are displayed as the relative ratio of Firefly luciferase activity to Renilla luciferase activity. C, EZH2 protein levels in HeLa cells after co‐transfection of pcDNA‐LINC01535 or pcDNA and miR‐214 mimics or miR‐NC. D, EZH2 protein levels in HeLa cells after transfection of sh‐LINC01535 or sh‐NC. E, ChIP assays were performed in HeLa cells using EZH2 specific antibody or non‐specific IgG. The enriched DNA was detected by qRT‐PCR with primers specific for the promoter of *miR‐214*. F, After transfection of EZH2 overexpression plasmid or empty plasmid into HeLa cells, EZH2 protein expression level and miR‐214 expression level was detected by Western blot and qRT‐PCR, respectively. G, After transfection of EZH2 specific shRNA or negative control shRNA into HeLa cells, EZH2 protein expression level and miR‐214 expression level was detected by Western blot and qRT‐PCR, respectively. H, After co‐transfection of pcDNA‐LINC01535 or pcDNA and sh‐EZH2 or sh‐NC into HeLa cells, miR‐214 expression level was detected by qRT‐PCR. I, After transfection of sh‐LINC01535 or sh‐NC into HeLa cells, miR‐214 expression level was detected by qRT‐PCR. Results are shown as mean ± SD of three independent experiments. **P < 0.01, ***P < 0.001, ns, not significant, by Student's *t* test (B, E, F, G, I) or one‐way ANOVA followed by Dunnett's multiple comparison test (A, H)

Many previous reports including ours have identified EZH2 as a critical target of miR‐214 in various cancers including cervical cancer.^34^, ^44^ Thus, we further investigated the effects of LINC01535 on EZH2. EZH2 3’UTR containing miR‐214 target site was cloned into luciferase reporter pmirGLO. Dual luciferase reporter assays displayed that enhanced expression of LINC01535 increased the luciferase activity of constructed reporter but not empty reporter (Figure 2A). The increase of luciferase activity was abolished by concurrent miR‐214 overexpression (Figure 2A). Reciprocally, inhibition of LINC01535 decreased the luciferase activity of constructed reporter but not empty reporter (Figure 2B). Western blot assays displayed that enhanced expression of LINC01535 up‐regulated EZH2 protein level (Figure 2C). The up‐regulation of EZH2 protein level was abolished by concurrent miR‐214 overexpression (Figure 2C). Reciprocally, inhibition of LINC01535 down‐regulated EZH2 protein level (Figure 2D). The interaction between lncRNA PVT1 and EZH2 has been reported to repress miR‐214 in ovarian cancer.^45^ Thus, we further investigated whether EZH2 also regulates miR‐214 in cervical cancer. ChIP assays revealed that EZH2 effectively bound to the promoter of *miR‐214* (Figure 2E). qRT‐PCR revealed that enhanced expression of EZH2 reduced miR‐214 expression level (Figure 2F). Inhibition of EZH2 increased miR‐214 expression level (Figure 2G). Next, we further investigated whether LINC01535 modulates miR‐214 expression via up‐regulating EZH2. qRT‐PCR revealed that enhanced expression of LINC01535 reduced miR‐214 expression level (Figure 2H). The reduction of miR‐214 expression level was abolished by concurrent inhibition of EZH2 (Figure 2H). Reciprocally, inhibition of LINC01535 up‐regulated miR‐214 expression level (Figure 2I). Collectively, these results demonstrated that LINC01535 represses the miR‐214/EZH2 regulatory loop, down‐regulates miR‐214 expression and up‐regulates EZH2 expression.

### 3.3 Enhanced expression of LINC01535 promotes cervical cancer cell growth, migration and invasion

**Figure 3:**
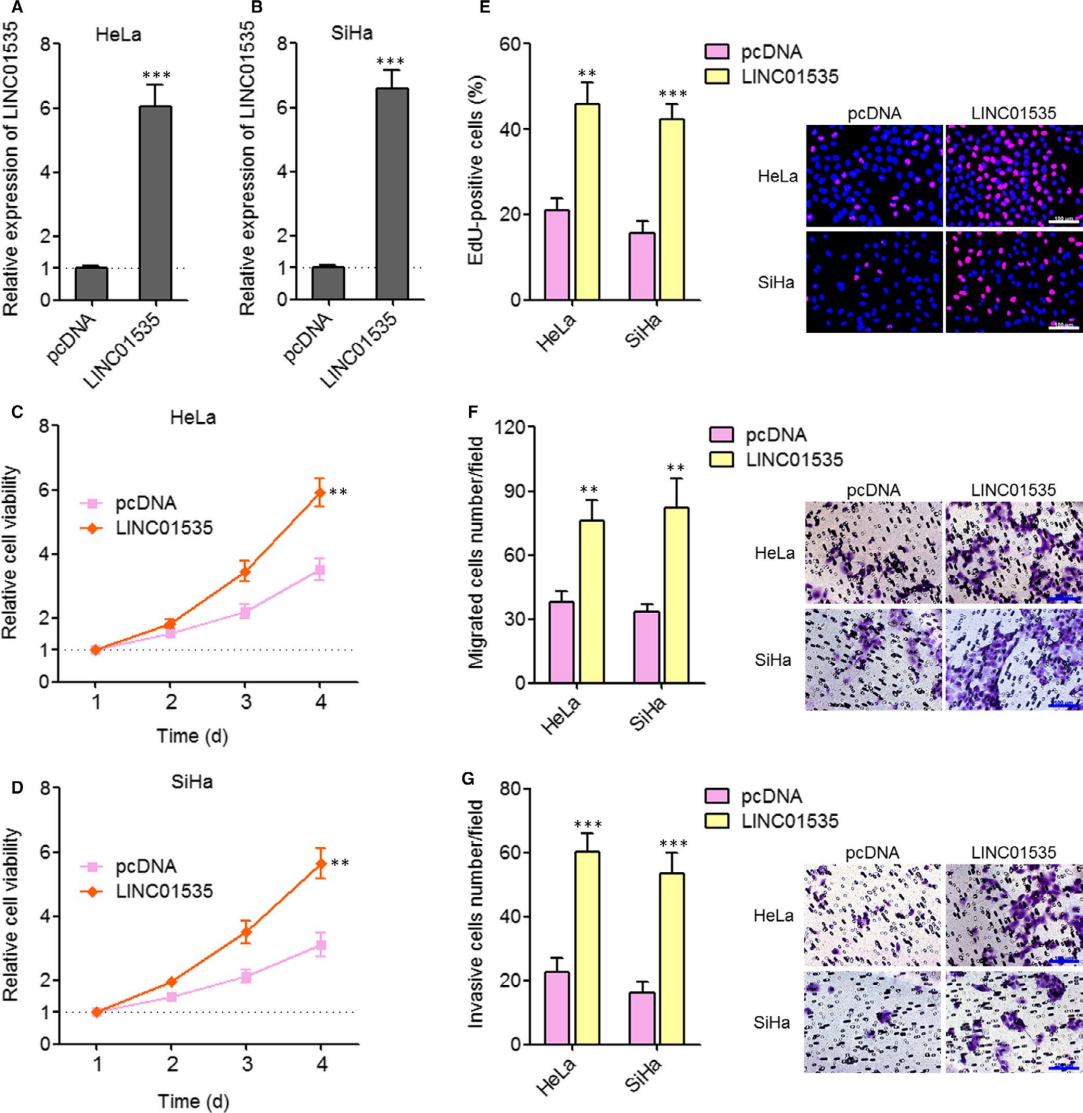
Enhanced expression of LINC01535 promotes cervical cancer cell growth, migration and invasion. A, LINC01535 expression in LINC01535 stably overexpressed and control HeLa cells was detected by qRT‐PCR. B, LINC01535 expression in LINC01535 stably overexpressed and control SiHa cells were detected by qRT‐PCR. C, Cell viabilities of LINC01535 stably overexpressed and control HeLa cells were evaluated by Glo cell viability assay. D, Cell viabilities of LINC01535 stably overexpressed and control SiHa cells were evaluated by Glo cell viability assay. E, Cell growth of LINC01535 stably overexpressed and control HeLa and SiHa cells was evaluated by ethynyl deoxyuridine staining. Scale bars, 100 µm. F, Cell migration of LINC01535 stably overexpressed and control HeLa and SiHa cells was evaluated by transwell migration assays. Scale bars, 100 µm. G, Cell invasion of LINC01535 stably overexpressed and control HeLa and SiHa cells was evaluated by transwell invasion assays. Scale bars, 100 µm. Results are shown as mean ± SD of three independent experiments. **P < 0.01, ***P < 0.001 by Student's *t* test

Several previous reports, including ours have revealed the tumour suppressive roles of miR‐214 and the oncogenic roles of EZH2 in cervical cancer.^34^, ^46^ Thus, we further investigated the biological roles of LINC01535 in cervical cancer. We constructed LINC01535 stably overexpressed HeLa and SiHa cells. The overexpression efficiencies were determined by qRT‐PCR (Figure 3A, 3B). Glo cell viability experiments showed that enhanced expression of LINC01535, up‐regulated cell viabilities of both HeLa and SiHa cells (Figure 3C, 3D). EdU staining experiments showed that enhanced expression of LINC01535 promoted cell proliferation of both HeLa and SiHa cells (Figure 3E). Transwell migration assays showed that enhanced expression of LINC01535 promoted cell migration of both HeLa and SiHa cells (Figure 3F). Transwell invasion assays showed that enhanced expression of LINC01535 promoted cell invasion of both HeLa and SiHa cells (Figure 3G). Therefore, these findings suggested that enhanced expression of LINC01535 promotes cervical cancer cell growth, migration and invasion.

### 3.4 Inhibition of LINC01535 suppresses cervical cancer cell growth, migration and invasion

**Figure 4:**
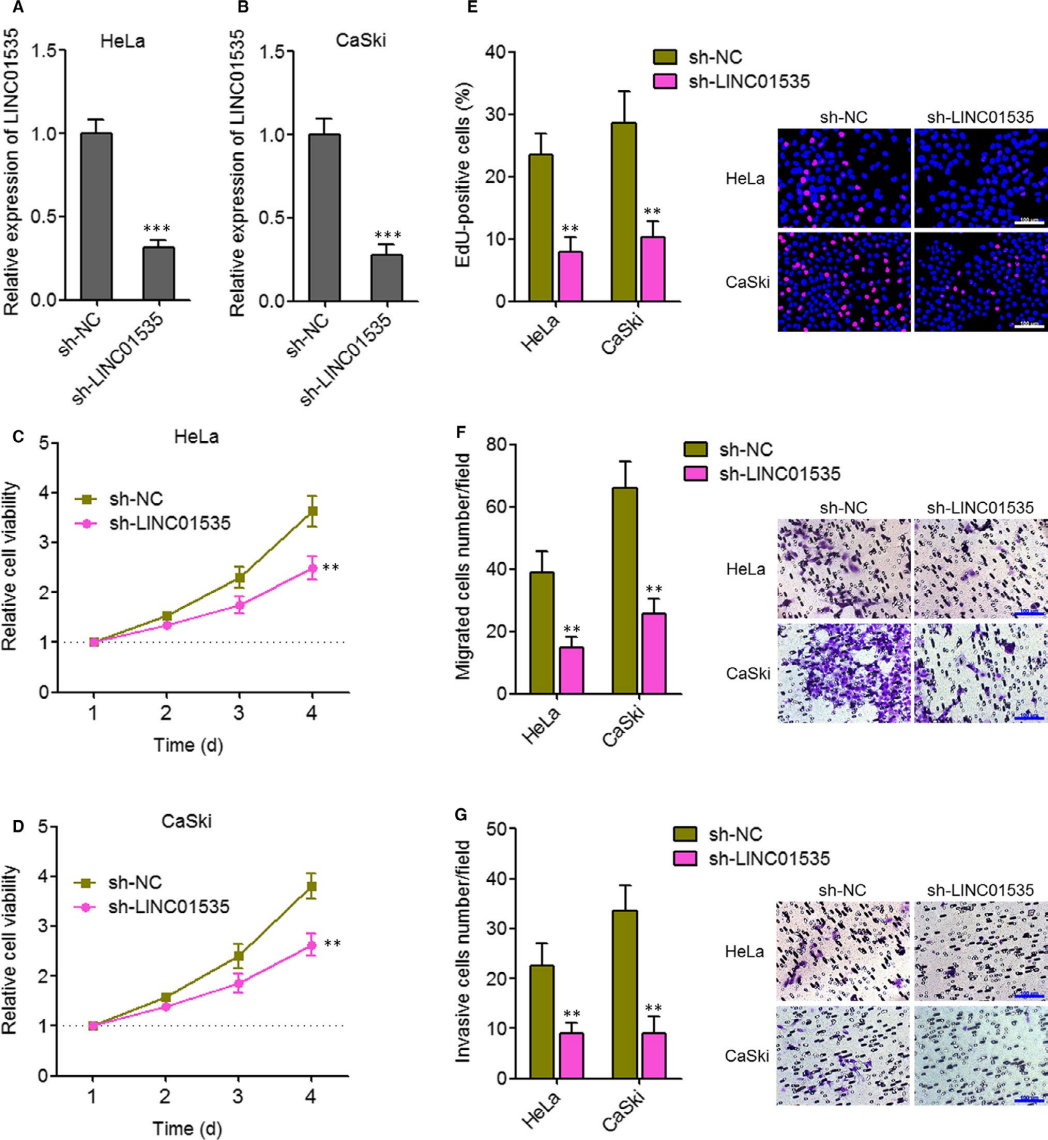
Inhibition of LINC01535 suppresses cervical cancer cell growth, migration and invasion. A, LINC01535 expression in LINC01535 stably silenced and control HeLa cells was detected by qRT‐PCR. B, LINC01535 expression in LINC01535 stably silenced and control CaSki cells was detected by qRT‐PCR. C, Cell viabilities of LINC01535 stably silenced and control HeLa cells were evaluated by Glo cell viability assay. D, Cell viabilities of LINC01535 stably silenced and control CaSki cells were evaluated by Glo cell viability assay. E, Cell growth of LINC01535 stably silenced and control HeLa and CaSki cells was evaluated by ethynyl deoxyuridine staining. Scale bars, 100 µm. F, Cell migration of LINC01535 stably silenced and control HeLa and CaSki cells was evaluated by transwell migration assays. Scale bars, 100 µm. G, Cell invasion of LINC01535 stably silenced and control HeLa and CaSki cells was evaluated by transwell invasion assays. Scale bars, 100 µm. Results are shown as mean ± SD of three independent experiments. **P < 0.01, ***P < 0.001 by Student's *t* test

Next, we further investigated the implications of targeting LINC01535 for cervical cancer. We constructed LINC01535 stably silenced HeLa and CaSki cells. The knock‐down efficiencies were determined by qRT‐PCR (Figure 4A, 4B). Glo cell viability experiments showed that inhibition of LINC01535 reduced cell viabilities of both HeLa and CaSki cells (Figure 4C, 4D). EdU staining experiments showed that inhibition of LINC01535 suppressed cell proliferation of both HeLa and CaSki cells (Figure 4E). Transwell migration assays showed that inhibition of LINC01535 suppressed cell migration of both HeLa and CaSki cells (Figure 4F). Transwell invasion assays showed that inhibition of LINC01535 suppressed cell invasion of both HeLa and CaSki cells (Figure 4G). Therefore, these findings suggested that inhibition of LINC01535 suppresses cervical cancer cell growth, migration and invasion.

### 3.5 Activation of the miR‐214/EZH2 regulatory loop reverses the roles of LINC01535 on cervical cancer cell growth, migration and invasion

**Figure 5:**
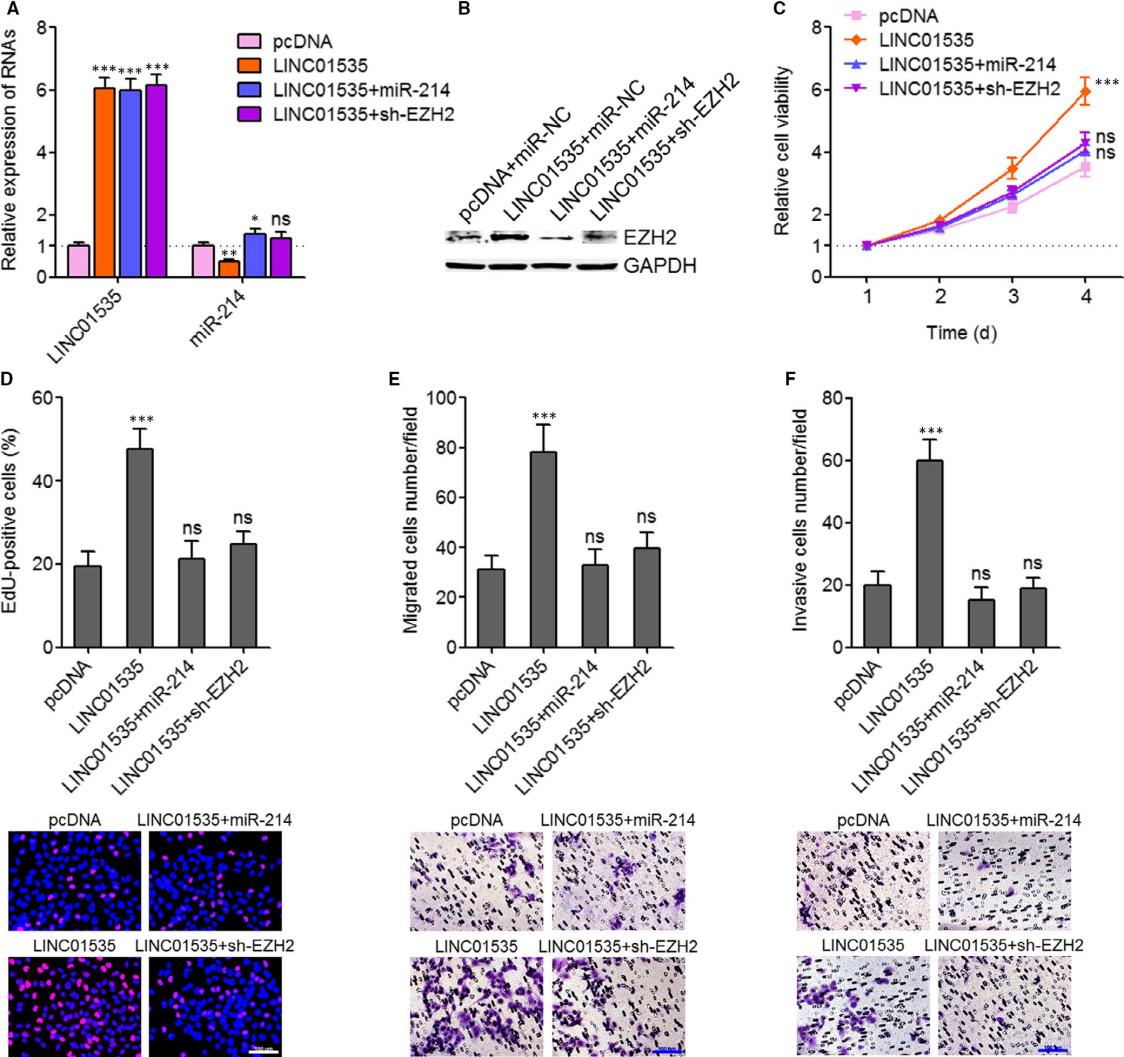
Activation of the miR‐214/EZH2 regulatory axis reverses the roles of LINC01535 on cervical cancer cell growth, migration and invasion. A, LINC01535 and miR‐214 expression in LINC01535 and miR‐214 concurrently overexpressed, LINC01535 overexpressed and concurrently EZH2 silenced, and control HeLa cells was detected by qRT‐PCR. B, EZH2 protein expression in LINC01535 and miR‐214 concurrently overexpressed, LINC01535 overexpressed and concurrently EZH2 silenced, and control HeLa cells was detected by wWestern blot. C, Cell viabilities of LINC01535 and miR‐214 concurrently overexpressed, LINC01535 overexpressed and concurrently EZH2 silenced, and control HeLa cells were evaluated by Glo cell viability assay. D, Cell growth of LINC01535 and miR‐214 concurrently overexpressed, LINC01535 overexpressed and concurrently EZH2 silenced, and control HeLa cells was evaluated by ethynyl deoxyuridine staining. Scale bars, 100 µm. E, Cell migration of LINC01535 and miR‐214 concurrently overexpressed, LINC01535 overexpressed and concurrently EZH2 silenced, and control HeLa cells was evaluated by transwell migration assays. Scale bars, 100 µm. F, Cell invasion of LINC01535 and miR‐214 concurrently overexpressed, LINC01535 overexpressed and concurrently EZH2 silenced, and control HeLa cells was evaluated by transwell invasion assays. Scale bars, 100 µm. Results are shown as mean ± SD of three independent experiments. *P < 0.05, **P < 0.01, ***P < 0.001, ns, not significant, by one‐way ANOVA followed by Dunnett's multiple comparison test

To determine whether the oncogenic roles of LINC01535 in cervical cancer are dependent on the repression of miR‐214/EZH2 regulatory loop, we stably overexpressed miR‐214 or stably silenced EZH2 in LINC01535 stably overexpressed HeLa cells (Figure 5A, 5B). The results further showed that although LINC01535 down‐regulated miR‐214 and up‐regulated EZH2, neither overexpression of miR‐214 nor silencing of EZH2 modulated the expression of LINC01535. Glo cell viability experiments showed that the increase of cell viability caused by LINC01535 overexpression was reversed by miR‐214 overexpression or EZH2 silencing (Figure 5C). EdU staining experiments showed that the promotion of cell proliferation caused by LINC01535 overexpression was reversed by miR‐214 overexpression or EZH2 silencing (Figure 5D). Transwell migration assays showed that the increase of cell migration caused by LINC01535 overexpression was reversed by miR‐214 overexpression or EZH2 silencing (Figure 5E). Transwell invasion assays showed that the increase of cell invasion caused by LINC01535 overexpression was reversed by miR‐214 overexpression or EZH2 silencing (Figure 5F). Therefore, these findings suggested that activation of the miR‐214/EZH2 regulatory loop reverses the roles of LINC01535 on cervical cancer cell growth, migration and invasion.

### 3.6 LINC01535 promotes cervical cancer growth in vivo via repressing the miR‐214/EZH2 regulatory loop

**Figure 6:**
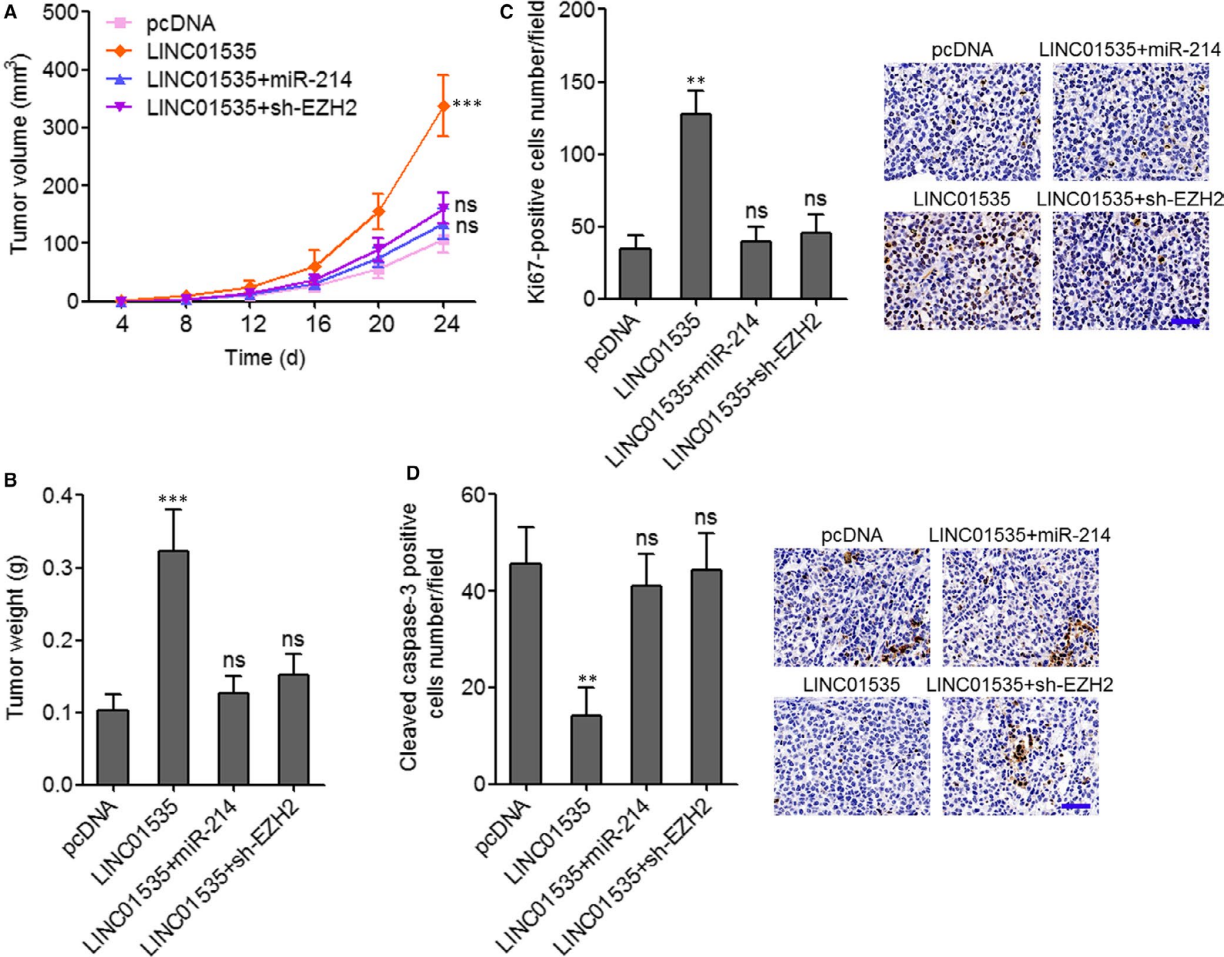
LINC01535 promotes cervical cancer growth in vivo via repressing the miR‐214/EZH2 regulatory axis. A, LINC01535 and miR‐214 concurrently overexpressed, LINC01535 overexpressed and concurrently EZH2 silenced, and control HeLa cells were subcutaneously injected into nude mice. Tumour volumes were measured every 4 days. B, Subcutaneous tumour weights were measured at the 24th day after injection. C, Ki67 immunohistochemistry (IHC) staining of subcutaneous tumours derived from B. Scale bars, 50 μm. D, Cleaved caspase‐3 IHC staining of subcutaneous tumours derived from B. Scale bars, 50 μm. Results are shown as mean ± SD of *n* = 6 mice in each group. **P < 0.01, ***P < 0.001, ns, not significant, by Kruskal‐Wallis test followed by Dunnett's multiple comparison test

To further investigate the roles of LINC01535/miR‐214/EZH2 regulatory loop in cervical cancer growth in vivo, LINC01535 and miR‐214 concurrently stably overexpressed, LINC01535 stably overexpressed and concurrently EZH2 stably silenced, and control HeLa cells were subcutaneously inoculated into nude mice. Subcutaneous tumour volumes were measured every 4 days and the subcutaneous tumours were resected and weighed on the 24th day after inoculation. As displayed in Figure 6A, 6B, enhanced expression of LINC01535 promoted tumour growth in vivo. The promotion of subcutaneous tumour growth caused by LINC01535 overexpression was reversed by miR‐214 overexpression or EZH2 silencing. Furthermore, proliferation marker Ki67 IHC staining of these subcutaneous tumours displayed that enhanced expression of LINC01535 promoted HeLa cell proliferation in vivo, which was reversed by miR‐214 overexpression or EZH2 silencing (Figure 6C). Apoptosis marker cleaved caspase‐3 IHC staining of these subcutaneous tumours displayed that enhanced expression of LINC01535 suppressed HeLa cell apoptosis in vivo, which was also reversed by miR‐214 overexpression or EZH2 silencing (Figure 6D). Therefore, these findings suggested that LINC01535 promotes cervical cancer growth in vivo via repressing the miR‐214/EZH2 regulatory loop.

### 3.7 LINC01535 is up‐regulated and negatively associated with the miR‐214/EZH2 regulatory loop in cervical cancer

**Figure 7:**
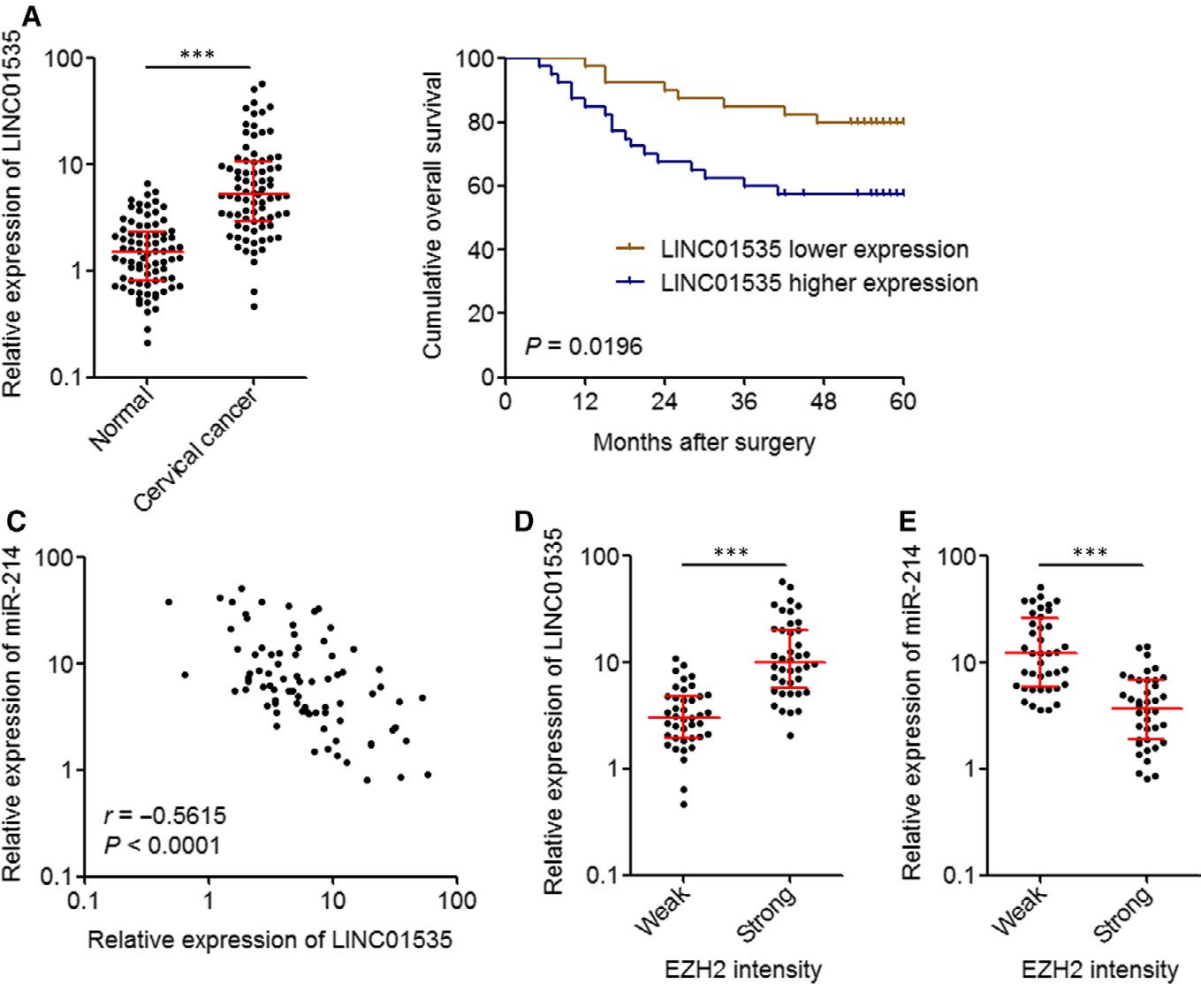
LINC01535 is up‐regulated and negatively associated with the miR‐214/EZH2 regulatory axis in cervical cancer. A, LINC01535 expression in 80 pairs of cervical cancer tissues and matched adjacent normal tissues was measured by qRT‐PCR. Results are shown as median with interquartile range. ***P < 0.0001 by Wilcoxon signed‐rank test. B, Kaplan‐Meier analysis of the correlation between LINC01535 expression level and overall survival of these 80 cervical cancer cases. The median expression level of LINC01535 was used as the cutoff. *P* = 0.0196 by log‐rank test. C, The correlation between LINC01535 and miR‐214 expression levels in these 80 cervical cancer tissues. *r* = −0.5615, *P* < 0.0001 by Pearson correlation analysis. D, LINC01535 expression levels in 80 cervical cancer tissues with strong or weak EZH2 staining intensity. The median EZH2 staining intensity was used as the cutoff. ***P < 0.0001 by Mann‐Whitney test. E, miR‐214 expression levels in 80 cervical cancer tissues with strong or weak EZH2 staining intensity. The median EZH2 staining intensity was used as the cutoff. ***P < 0.0001 by Mann‐Whitney test

To explore whether the LINC01535/miR‐214/EZH2 regulatory axis exists in clinical tissue specimens, we collected 80 pairs of cervical cancer tissues and matched adjacent normal cervical tissues. The expression of LINC01535 in these tissue specimens was detected by qRT‐PCR. As displayed in Figure 7A, LINC01535 was markedly up‐regulated in cervical cancer tissues compared with paired normal tissues. The analysis of the correlation between LINC01535 and the clinicopathological characteristics in these 80 cervical cancer cases presented that LINC01535 high expression was correlated with advanced FIGO stage (*P* = 0.036) and lymph node metastasis (*P* = 0.045) (Table 1). Kaplan‐Meier analyses in these 80 cervical cancer cases presented that increased LINC01535 high expression was correlated with worse overall survival (*P* = 0.0196) (Figure 7B). We next analysed the correlation between LINC01535 and miR‐214/EZH2 expression in cervical cancer tissues. Consistent with the down‐regulation of miR‐214 by LINC01535, the expression of miR‐214 was significantly reversely correlated with that of LINC01535 in cervical cancer tissues (r = −0.5615, *P* < 0.0196) (Figure 7C). IHC staining of EZH2 displayed that the cervical cancer tissues with strong EZH2 staining intensity had higher LINC01535 expression and lower miR‐214 expression than those of cervical cancer tissues with weak EZH2 staining intensity (Figure 7D, 7E). Therefore, these findings suggested that LINC01535 is up‐regulated in cervical cancer tissues and correlated with advanced clinical stage and poor prognosis of cervical cancer patients. Moreover, the expression of LINC01535 is negatively correlated with the expression of miR‐214/EZH2 regulatory loop in cervical cancer.

**Table 1:** Correlation between LINC01535 expression and clinicopathological characteristics in 80 cases of cervical cancer

Characteristics	N	LINC01535 expression		*P* value
		Low	High	
Age (years)				
≥45	53	25	28	0.478
<45	27	15	12	
Histology				
Squamous	55	26	29	0.469
Adenocarcinoma	25	14	11	
Tumour size (cm)				
≥4	29	12	17	0.245
<4	51	28	23	
FIGO stage				
I	51	30	21	0.036
II	29	10	19	
Lymph node metastasis				
Positive	22	7	15	0.045
Negative	58	33	25	

*P* values were calculated by Pearson chi‐square tests.

## 4 DISCUSSION

High throughput sequencings have identified significantly greater number of lncRNAs that mRNAs, with more than 58 000 lncRNAs and only about 21 000 mRNAs.^47^ Although some of these lncRNAs have been investigated in human diseases, including cervical cancer, clinical significances of most of these lncRNAs are unclear.^48^, ^49^, ^50^, ^51^, ^52^ In this study, we identified an oncogenic lncRNA LINC01535 in cervical cancer. Our data revealed that LINC01535 is significantly up‐regulated in cervical cancer tissues compared with adjacent normal cervical tissues. High expression of LINC01535 is associated with advanced FIGO stage, lymph node metastasis and poor survival of cervical cancer patients. Results of functional experiments indicated that enhanced expression of LINC01535 promotes cervical cancer cell growth, migration and invasion in vitro, and cervical cancer xenograft growth in vivo. Reciprocally, inhibition of LINC01535 suppresses cervical cancer cell growth, migration and invasion. Therefore, these findings suggest LINC01535 as on oncogene in cervical cancer and imply that LINC01535 may be a promising prognostic biomarker and therapeutic target for cervical cancer.

The identification of LINC01535 is dependent on its three miR‐214 binding sites accumulating in a very short region. The relative short region with relatively more miRNAs binding sites indicates more strong binding possibility.^53^ Using dual luciferase reporter assay and RNA pull‐down assay, we verified the specific interaction between LINC01535 and miR‐214. The interaction between LINC01535 and miR‐214 did not regulate the expression of LINC01535, as neither overexpression nor inhibition of miR‐214 changed the expression of LINC01535. But the interaction between LINC01535 and miR‐214 regulates the roles of miR‐214. Several previous reports, including ours, have identified EZH2 as a critical target of miR‐214.^34^, ^44^, ^54^ In the present study, we further found that enhanced expression of LINC01535 up‐regulates the luciferase activity of EZH2 3’UTR and the protein level of EZH2. Inhibition of LINC01535 down‐regulates the luciferase activity of EZH2 3’UTR and the protein level of EZH2. The up‐regulation of EZH2 3’UTR activity and protein level caused by LINC01535 can be abolished by concurrent overexpression of miR‐214.

Intriguingly, except the repression of EZH2 by miR‐214, in this study we also found that EZH2 directly binds the promoter of *miR‐214* and represses *miR‐214* expression. Thus, miR‐214 and EZH2 form double negative feedback regulatory loop. Via up‐regulating EZH2, LINC01535 represses *miR‐214* expression, and the repression of miR‐214 caused by LINC01535 can be abolished by silencing EZH2. Collectively, these findings demonstrated that LINC01535 binds miR‐214, relieves the repressive roles of miR‐214 on EZH2, therefore up‐regulates EZH2 expression, and further repressing miR‐214 expression via the up‐regulation of EZH2. LINC01535 modulates the miR‐214/EZH2 double negative feedback loop to lower miR‐214 and higher EZH2. Rescue assays showed that activation of the miR‐214/EZH2 regulatory loop either by overexpression of miR‐214 or by silencing of EZH2, both reverse the roles of LINC01535 in promoting cervical cancer cell growth, migration and invasion in vitro, and cervical cancer xenograft growth in vivo. In addition, the expression of LINC01535 is inversely associated with that of miR‐214 and positively associated with that of EZH2 in cervical cancer tissues, which support the negative regulation of miR‐214/EZH2 loop by LINC01535.

Except EZH2, HMGA1, GALNT7, Bcl2l2, PSMD10, Wnt/β‐catenin pathway and p53 pathway are reported downstream targets of miR‐214.^37^, ^38^, ^39^, ^55^, ^56^, ^57^ Through binding and sequestering miR‐214, LINC01535 may also regulate these genes and pathways. Further investigations of the effects of LINC01535 on these miR‐214 downstream targets can enhance the understanding of LINC01535 and the application of targeting LINC01535 for cervical cancer treatment.

In summary, this study identified an oncogenic lncRNA LINC01535 in cervical cancer. LINC01535 is increased and associated with poor prognosis in cervical cancer. LINC01535 promotes cervical cancer cell growth, migration and invasion in vitro and xenograft growth in vivo via repressing the miR‐214/EZH2 regulatory loop. Our findings suggest LINC01535 as a novel candidate for the prognosis and therapy of cervical cancer.

## CONFLICT OF INTEREST

The authors declare that they have no conflict of interest.

## AUTHORS' CONTRIBUTIONS

HS and YL designed the study; YL, XJ, YL, YY, LL, XW and GL performed the experiments; HS and YL collected and analyzed the data. HS wrote the manuscript. All authors read and approved the final manuscript.

## Data Availability

The data that support the findings of this study are available from the corresponding author upon reasonable request.
